# Bacterial Diversity and Community Structure in the Pine Wood Nematode *Bursaphelenchus xylophilus* and *B*. *mucronatus* with Different Virulence by High-Throughput Sequencing of the 16S rDNA

**DOI:** 10.1371/journal.pone.0137386

**Published:** 2015-09-15

**Authors:** Yang Xiang, Xiao-Qin Wu, Ai-Dong Zhou

**Affiliations:** 1 Co-Innovation Center for Sustainable Forestry in Southern China, College of Forestry, Nanjing Forestry University, Nanjing, Jiangsu, China; 2 Jiangsu Key Laboratory for Prevention and Management of Invasive Species, Nanjing Forestry University, Nanjing, Jiangsu, China; James Hutton Institute, UNITED KINGDOM

## Abstract

*Bursaphelenchus xylophilus* is the pathogen of pine wilt disease. *Bursaphelenchus mucronatus* is similar to *B*. *xylophilus* in morphology. Both species share a common niche, but they are quite different in pathogenicity. Presently, the role of bacteria in pine wilt disease development has been widely speculated. The diversity of bacteria associated with *B*. *xylophilus* and *B*. *mucronatus* with different virulence remains unclear. In this study, virulence of four *B*. *xylophilus* and four *B*. *mucronatus* strains were evaluated by inoculating *Pinus thunbergii*. High-throughput sequencing targeted 16S rDNA of different virulence nematode strains was carried out. The associated bacterial community structures of the eight strains were analyzed. The results showed that 634,051 high-quality sequences were obtained from the eight nematode strains. The number of OTUs of bacteria associated with *B*. *mucronatus* was generally greater than those of *B*. *xylophilus*. The richness of the community of bacteria associated with high virulent *B*. *xylophilus* ZL1 and AmA3 was higher than moderately virulent *B*. *xylophilus* AA3, HE2, and all *B*. *mucronatus* strains. While the diversity of bacteria associated with *B*. *mucronatus* was higher than *B*. *xylophilus*. *Stenotrophomonas*, Pseudomonadaceae_Unclassified or Rhizobiaceae_Unclassified were predominant in the nematode strains with different virulence. Oxalobacteraceae and *Achromobacter* were found more abundant in the low virulent *B*. *xylophilus* and non-virulent *B*. *mucronatus* strains.

## Introduction

Pine wilt disease (PWD) is a destructive disease caused by the pine wood nematode (PWN), *Bursaphelenchus xylophilus*. It is native to North America and has been spread to Japan, China, South Korea and Europe, subsequently causing huge economic losses and decimating ecological systems in these countries. *Bursaphelenchus mucronatus* is similar to *B*. *xylophilus* in morphology and distributed in Eastern Asia, Americas and Europe [1].

The onset of pine wilt disease is rapid and susceptible pines can be killed within a year [2]. As for the symptoms and infection rate after infection of *B*. *xylophilus*, there is a difference in virulence among different *B*. *xylophilus* strains. The strains are divided into highly virulent, moderately virulent and low virulent strains [3]. *B*. *mucronatus* has been found in dead pines in the areas without pine wilt nematode infection in China. The pathogenicity of *B*. *mucronatus* has attracted increasing attention [4–6]. Some studies suggested that *B*. *mucronatus* is not pathogenic [7–9], but more researchers opined that *B*. *mucronatus* had low virulence [10, 11], potential virulence [12] or a certain degree of virulence [13–15]. The pathogenicity of the nematodes is different according to the differences of the population sources, strains and developing stages of the nematodes, hosts, and temperature [16–18].

The pathogenesis of pine wilt disease is still unclear. Occurrence of the disease involves nematodes, insect vectors, bacteria, hosts and a number of other factors. Recent studies on the relationship between *B*. *xylophilus* and bacteria found that bacteria may play a role in pine wilt disease [11, 19–22]. Researches on species of bacteria associated with *B*. *xylophilus* have shown that *B*. *xylophilus* from different countries and regions carried different bacterial diversities [23, 24, 25]. The role of bacterial community in PWD has been studied [25–30]. New findings from *B*. *xylophilus* (transcriptomics and secretomics) also show the existence of bacterial putative effectors that may contribute for nematode pathogenicity in the tree [31–33]. However, little research has been done on the relationship between bacteria and *B*. *mucronatus* [34, 35]. At present, differences of bacteria associated with *B*. *xylophilus* and *B*. *mucronatus* and their relationships with the virulence of nematodes have not been reported.

Yuan et al. found endo-bacteria in *B*. *xylophilus* and *B*. *mucronatus* [36]. Wu et al. isolated 15 species of culturable endo-bacteria from *B*. *xylophilus* in China and found that *Stenotrophomonas maltophilia* and *Achromobacter xylosoxidans* subsp. *xylosoxidans* may relate to the differences in virulent of the nematode strains [26]. Traditional researches on bacterial diversity use culture-depended methods, but only less than 1% microorganisms in nature can be isolated [37]. Culture methods fail to determine overall situation of bacteria associated with *B*. *xylophilus* and are inadequate in analyzing relationship between virulence of *B*. *xylophilus* and bacteria. Molecular analysis of the microbial community associated with the nematodes isolated from *Pinus pinaster* was performed by Proença [38]. Tian identified 64 species of bacteria from two 16S rDNA clone libraries constructed from a Chinese and a Japanese *B*. *mucronatus* populations [35]. Culture-independent methods showed their advantage in analyzing the bacteria associated with nematodes. Therefore, identifications of the isolated strains obtained only by biochemical methods maybe incomplete [39].

This study used high-throughput sequencing targeted 16S rDNA to analyze diversity of bacteria associated with *B*. *xylophilus* and *B*. *mucronatus* in combination with virulence of different nematode strains on pine seedlings to better understand the diversity of bacteria from *B*. *xylophilus* and *B*. *mucronatus* with different virulence.

## Materials and Methods

### *Bursaphelenchus xylophilus* and *B*. *mucronatus* strains

Four *B*. *xylophilus* strains and four *B*. *mucronatus* strains with different virulence were selected. All of the nematode strains were deposited in Jiangsu Key Laboratory for Prevention and Management of Invasive Species, China. The origins and hosts of eight strains were listed in Table 1.

**Table 1:** *Bursaphelenchus xylophilus* and *B*. *mucronatus* strains for virulence test.

Nematodes	Nematode strains	Pine hosts	Year of collection	Sampling region
*B*. *xylophilus*	ZL1	*Pinus massoniana*	2004	Linhai, Zhejiang
	AmA3	*P*. *massoniana*	2004	Ma’anshan, Anhui
	HE2	*P*. *thunbergii*	2004	Enshi, Hubei
	AA3	*P*. *taiwanensis*	2004	Anqing, Anhui
*B*. *mucronatus*	CFS1	*P*. *elliottii*	2005	Fushun, Sichuan
	SD1	*P*. *massoniana*	2005	Dazhu, Sichuan
	JNL10	*P*. *taeda*	2004	Nanjing, Jiangsu
	GHB3	*P*. *massoniana*	2004	Huizhou, Guangdong

### Virulence test of the nematode strains

*Bursaphelenchus xylophilus* and *B*. *mucronatus* strains were cultured on a fungus *Botrytis cinerea* at 25°C for 7 days. The propagated nematodes were collected with a Baermann funnel and were rinsed three times with sterile deionized water. The nematode suspension was prepared with sterile deionized water.

Four-year-old *Pinus thunbergii* seedlings with a similar growing condition were used. *P*. *thunbergii* stems were cut 15 cm above the soil level with a sterilized scalpel. A piece of sterile absorbent cotton was placed on each wound and 200 μL nematodes suspension (about 5,000 individuals) was pipetted into wounds. Then the wounds were covered by parafilm. Control was *P*. *thunbergii* seedlings inoculated with 200 μL sterile deionized water. Each treatment had 5 replicates. The disease development of *P*. *thunbergii* seedlings was observed at an interval of two days. The infection rate is the prevalence of the disease. The disease severity of *P*. *thunbergii* seedlings was divided into five levels: 0, the seedlings healthy with green needles and growing well; I, a few needles turning brown; II, half of the needles turning brown and the terminal shoots of seedlings bending; III, most of the needles turning brown and dead and the terminal shoots of seedlings drooping; IV, all of the needles turning brown and the whole seedling wilt. The infection rates and the disease severity index were calculated according to Fang [40].

$$\text{Infection rates}=\frac{\sum \text{number of infected plants with symptoms}}{\text{Total number of plants}}\times 100\%$$

$$\text{Disease severity index}\;(\text{DSI})=\frac{\sum \text{number of disease plants}\times \text{symptom stage}}{\text{Total number of plants}\;\times \;\text{highest symptom stage}}\times 100$$

### Surface sterilization of nematodes

A Baermann funnel was used to extract the nematodes. The nematodes were washed three times with sterile water, followed by sterilization of shaking the nematodes in 1% mercury bichloride for 30 min. After that, the nematodes were washed additional three times with sterile water, followed by shaking in a mixture of 1% streptomycin sulphate and 1% gentamicin for 30 min. Then, the nematodes were washed three times with sterile water. The nematode suspension was centrifuged and the supernatant was discarded. The precipitate containing nematode was resuspended with 1 mL sterile water. One hundred μL of the nematode suspension (about 2,500 individuals) was poured onto NA Petri dishes [26]. The plates were incubated at 28°C for 48 h to check presence of any bacterial colonies.

### DNA extraction and PCR amplification of bacteria associated with nematodes

Based on our preliminary research (unpublished data), 50,000 individual nematodes would be sufficient for DNA extraction and suitable for subsequent high throughput sequencing (e.g: concentration >50ng/μL, OD260/280 ranging from 1.8 to 2.0, and total amount >5000ng). The surfaces of nematodes were checked to be aseptic and 50,000 individual nematodes were ground in liquid nitrogen with a sterile mortar and a pestle. The ground nematodes were transferred to a 1.5 mL centrifuge tube and the total genomic DNA (PWN+PWN-associated bacteria) was extracted by E.Z.N.A. Mullusc DNA Kit (Norcross, OMEGA, USA).

To study the diversity and composition of bacteria associated with nematodes, a pair of PCR primers 515f/806r targeting bacterial 16S rRNA V4 region was applied in PCR amplification [41]. To distinguish the different samples, a Barcoded-tag with six nucleotide bases was randomly added to the upstream of the universal primer. The primers which added with Barcoded-tag sequences were Barcoded-tag fusion primers (BFP). After quantification and quality control, PCR products were gradually diluted and quantified. 16S rDNA PCR products were sequenced using 300bp paired-end model with the MiSeq system (Illumina, USA). The EzTaxon-e database (http://eztaxon-e.ezbiocloud.net/) was used for bacterial taxonomic identification.

### Bioinformatic analysis

The lengths of short reads were extended by finding the overlap between paired-end reads by the FLASH software [41]. Low quality data were filtered out using QIIME software [42]. The reads were sorted according to barcode sequences and the sample sources. The number of reads of each sample was counted. Sequences were clustered to operational taxonomic unit (OTU) at 97% sequence similarity by using UPARSE software [43].

To calculate the Alpha diversity, species richness (Chao), species coverage (Coverage, *C*) and species diversity (Simpson's diversity index, *1-D* / Shannon-Wiener Index, *H*) were calculated. Python script alpha_rarefaction.py from QIIME—1.7.0 was used for diversity index analysis with default parameter setting. The richness index Chao, was used to estimate the number of OTUs in the bacterial communities. Simpson and Shannon indexes were used to estimate the diversity of the OTUs. Community structure analyses were based on the phylum and genus taxonomy levels.

### Nucleotide Sequence Accession Numbers

Nucleotide Sequences were deposited at NCBI SRA database under the accession numbers SRX876433 and SRX876463-SRX876469.

## Results

### Pathogenicity of *B*. *xylophilus* and *B*. *mucronatus* on *P*. *thunbergii*

*P*. *thunbergii* seedlings of all treatments had a similar growing condition. After inoculated with nematodes, the *P*. *thunbergii* seedlings showed differences in symptoms. After 20 DAI (days after inoculation), infection rates of *P*. *thunbergii* inoculated with *B*. *xylophilus* strains ZL1, AmA3, AA3 and HE2 were 100%, 80%, 80% and 60%, respectively. The infection rates of *P*. *thunbergii* inoculated with *B*. *mucronatus* strains CFS1 and SD1 were 40% and 20%, respectively. Thus, six nematode strains were able to infect and induce wilting symptoms. No symptom was found when inoculated *P*. *thunbergii* seedlings with *B*. *mucronatus* strains GHB3 and JNL10. On the 30 DAI, *P*. *thunbergii* seedlings inoculated with the four *B*. *xylophilus* strains and *B*. *mucronatus* strains CFS1 and SD1 all showed symptoms. While the disease severity index of *P*. *thunbergii* seedlings were different. All of the *P*. *thunbergii* seedlings which inoculated with four *B*. *xylophilus* strains were wilted in 40 DAI, and all of the disease severity index were 100 (Table 2). The wilting process of *P*. *thunbergii* seedlings after inoculated with nematodes was different. Wilt symptoms were observed when inoculated with *B*. *xylophilus* strains ZL1 and AmA3, and the first *P*. *thunbergii* seedling was dead in 24 days. The *P*. *thunbergii* seedlings started to die in the 31 and 33 days after inoculated with *B*. *xylophilus* strains AA3 and HE2, respectively. It indicated that ZL1 and AmA3 caused death to *P*. *thunbergii* seedlings at a faster rate than AA3 and HE2. All of the *P*. *thunbergii* seedlings were infected when inoculated with *B*. *mucronatus* strains CFS1 and SD1, but no seedling was dead. Therefore, the pathogenicity of CFS1 and SD1 was weaker than those of other four *B*. *xylophilus* strains. A mild wilt symptom was observed after inoculated with *B*. *mucronatus* strain JNL10, and the infection index was 5. No wilt symptom was observed after inoculated with *B*. *mucronatus* strain GHB3 (Table 2). Thus, JNL10 showed rather weak pathogenicity, while GHB3 was not pathogenic to *P*. *thunbergii*. In summary, *B*. *xylophilus* strains ZL1 and AmA3 were highly virulent (HV) and strains AA3 and HE2 were moderately virulent (MV) to *P*. *thunbergii*. *B*. *mucronatus* strains CFS1 and SD1 were moderately virulent (MV), and strain JNL10 had low virulence (LV) to *P*. *thunbergii*, and the GHB3 strain was not virulent (NV).

**Table 2:** The symptoms of *P*. *thunbergii* after inoculated with *B*. *xylophilus* and *B*. *mucronatus*.

	Infection rates/ %			DSI				
Nematode strains	20^th^ day	30^th^ day	40^th^ day	20^th^ day	30^th^ day	40^th^ day	Days of symptoms appeared (d)	Days of *P*. *thunbergii* wilted (d)
ZL1	100	100	100	30	85	100	11	24
AMA3	80	100	100	35	70	100	11	24
AA3	80	100	100	25	45	100	13	31
HE2	60	100	100	20	45	100	17	33
CFS1	40	100	100	20	45	75	19	-
SD1	20	100	100	10	35	60	19	-
JNL10	0	0	20	0	0	5	31	-
GHB3	0	0	0	0	0	0	-	-
CK	0	0	0	0	0	0	-	-

### Species abundance analysis of bacteria associated with *B*. *xylophilus* and *B*. *mucronatus*

**Fig 1:**
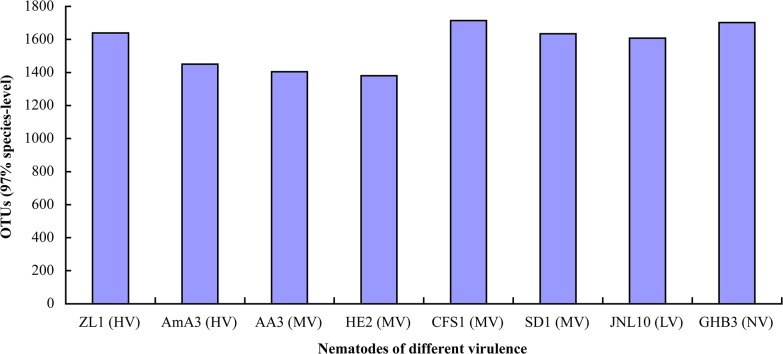
OTU numbers of bacteria associated with *B*. *xylophilus* and *B*. *mucronatus*.

Sequences of the bacteria associated with *B*. *xylophilus* and *B*. *mucronatus* with different virulence were filtered using QIIME software. The statistical results showed that 634,051 high-quality sequences were obtained from the eight nematode strains. The average sequencing results of the eight samples were 79,256. The average length of the assembled reads was 252 bp. The OTUs for ZL1, AmA3, AA3 and HE2 were 1639, 1450, 1405 and 1380, respectively. The OTUs for CFS1, SD1, JNL10 and GHB3 were 1714, 1635, 1608 and 1702, respectively. The HE2 strain contained the smallest number of OTUs, while CFS1 contained the most abundant OTUs. The number of OTUs of bacteria associated with *B*. *mucronatus* was generally greater than those of *B*. *xylophilus* (Fig 1).

**Fig 2:**
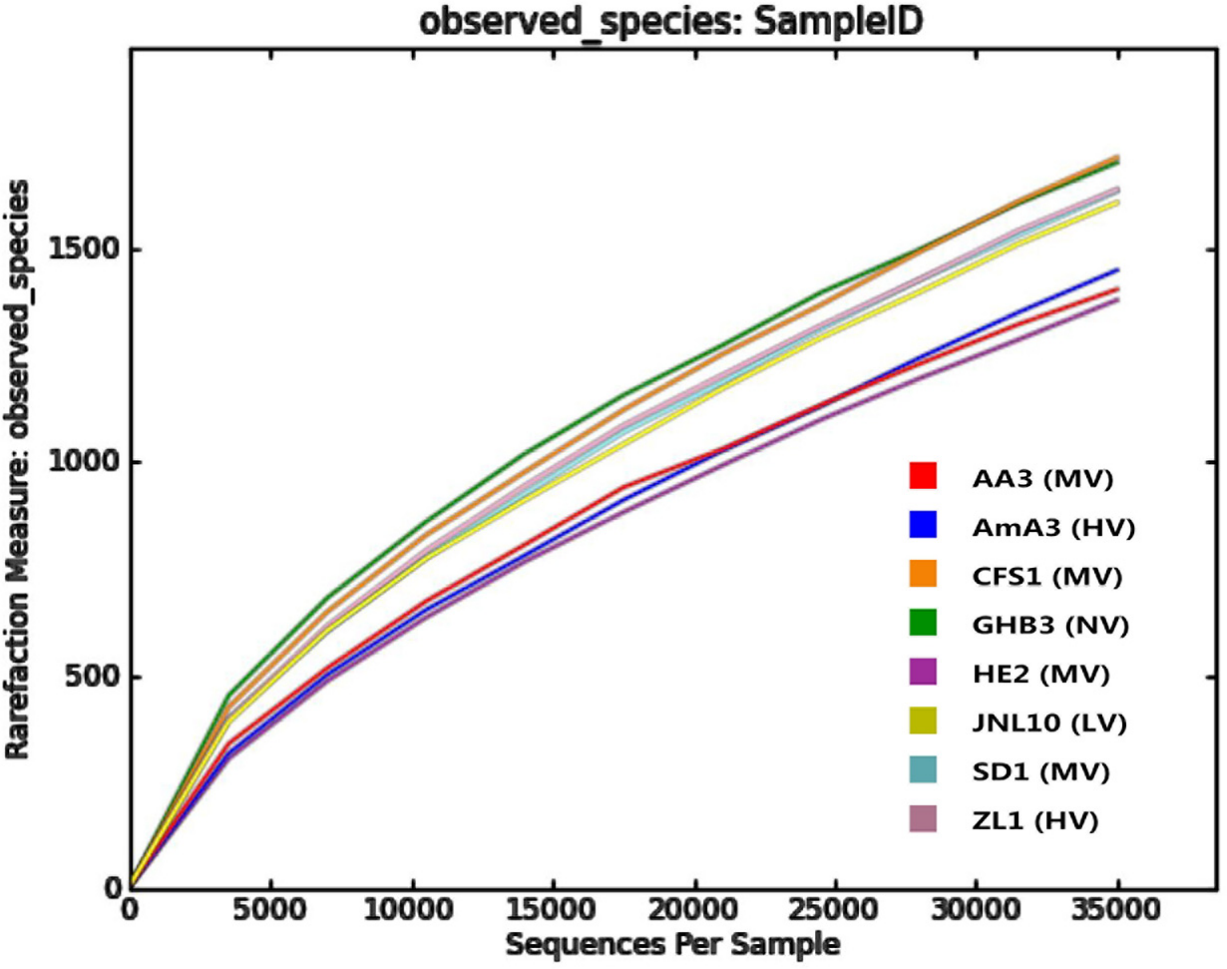
Rarefaction analysis of bacteria associated with *B*. *xylophilus* and *B*. *mucronatus*.

The rarefaction curves of all nematode-bacteria communities were flat and stable but did not reach maximum (Fig 2). It indicated that the sampling method was reasonable, reliable and able to represent the actual bacteria communities but a very small number of bacteria still remain undetected.

All of the coverage estimations were 100 of the bacteria associated with *B*. *xylophilus* and *B*. *mucronatus* with different virulence. The results indicated a high coverage of the sequencing libraries, and showed the diversity of the associated bacteria. Chao indexes showed that the richness of the community of bacteria associated with highly virulent *B*. *xylophilus* ZL1 and AmA3 was higher than moderately virulent *B*. *xylophilus* AA3, HE2, and all *B*. *mucronatus* strains. The Simpson and Shannon indexes of bacteria associated with *B*. *mucronatus* were both higher than those with *B*. *xylophilus*, indicating that diversity of bacteria associated with *B*. *mucronatus* was higher than those with *B*. *xylophilus* (Table 3).

**Table 3:** The diversity index of bacteria associated with *B*. *xylophilus* and *B*. *mucronatus*.

Nematodes	Nematode strains	Coverage (*C*) / %	Chao (97%)	Simpson (*1-D*) (97%)	*Shannon* (*H*) (97%)
*B*. *xylophilus*	ZL1 (HV)	100	7289	0.769	4.126
	AmA3 (HV)	100	7276	0.871	4.995
	AA3 (MV)	100	4098	0.848	5.121
	HE2 (MV)	100	4663	0.829	4.552
*B*. *mucronatus*	CFS1 (MV)	100	6319	0.919	6.140
	SD1 (MV)	100	5967	0.898	5.602
	JNL10 (LV)	100	6743	0.896	5.588
	GHB3 (NV)	100	4786	0.933	6.132

### Community structure analysis of bacterial associated with *B*. *xylophilus* and *B*. *mucronatus* at phylum level

**Fig 3:**
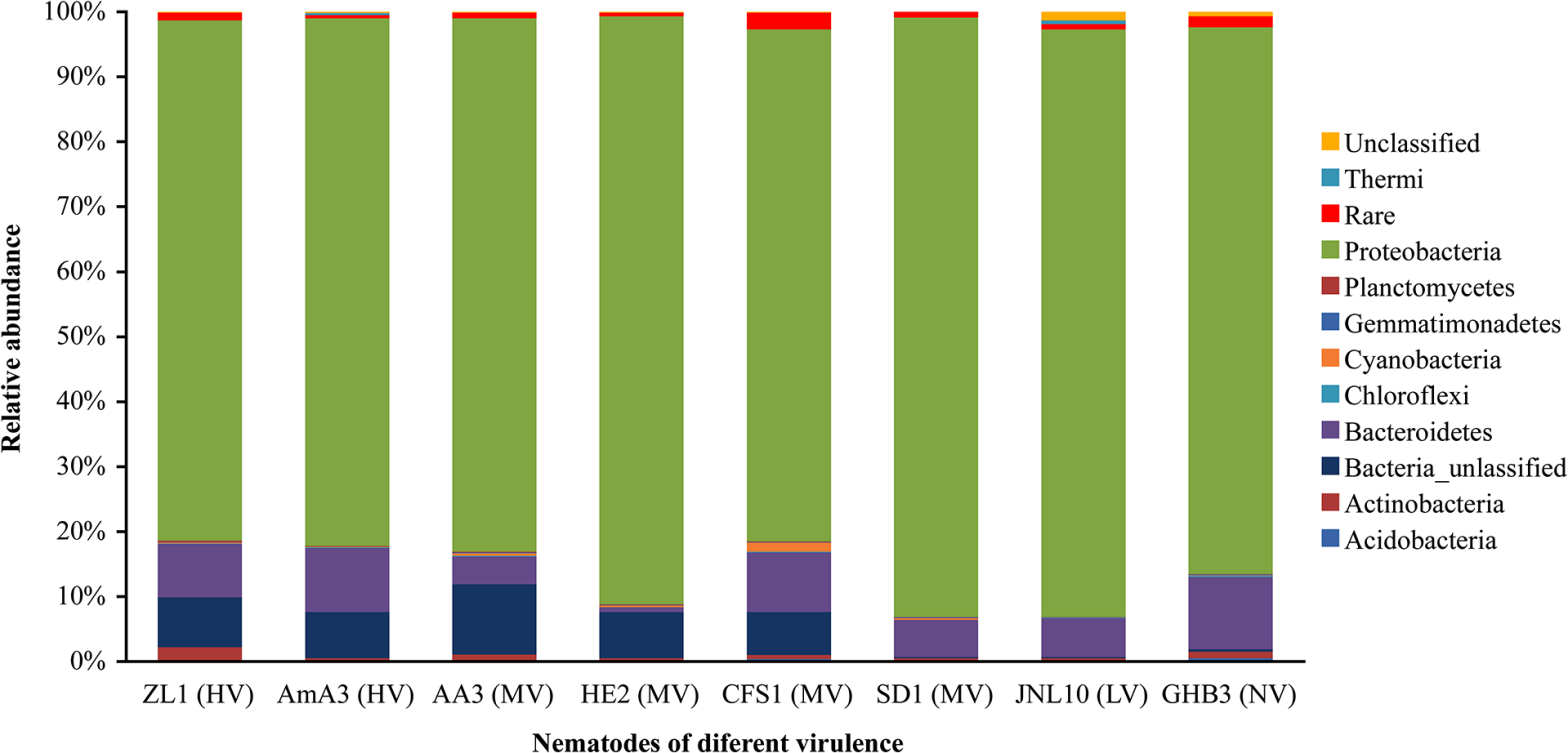
The bacterial composition of *B*. *xylophilus* and *B*. *mucronatus* at phylum level.

The OTUs of bacteria associated with the nematodes were classified and organized. At phylum level, bacteria associated with the eight strains of nematodes were classified to 9–12 taxonomic phyla. The bacteria associated with different nematode strains were mainly Proteobacteria, Bacteroidetes, Acidobacteria, Actinobacteria, Chloroflexi, Cyanobacteria, Firmicutes, Gemmatimonadetes, Planctomycetes and Verrucomicrobia. Proteobacteria was the most predominant phylum in the eight libraries, followed by Bacteroidetes. Some OTUs were not identified as phylum in the sequencing results. For the bacteria associated with *B*. *xylophilus*, the proportion of unidentified OTUs was more than 7%. Unidentified OTUs of bacteria was 6.6% in *B*. *mucronatus* strain CFS1, and the abundance was less than 0.4% in *B*. *mucronatus* strain SD1, JNL10 and GHB3 (Fig 3).

### Community structures of bacteria associated with *B*. *xylophilus* and *B*. *mucronatus* at genus level

There are at least 24 groups of bacteria associated with *B*. *xylophilus* and *B*. *mucronatus* at genus level by 16S rDNA high-throughput sequencing.

#### Predominant bacteria associated with *B*. *xylophilus*

Bacteria associated with *B*. *xylophilus* were sorted by their abundance. The abundance higher than 10% were the predominant bacteria associated with the nematode strains. As shown in Table 4, the predominant bacteria associated with the highly virulent *B*. *xylophilus* ZL1 were Pseudomonadaceae_Unclassified, *Pseudomonas* and *Stenotrophomonas*. The predominant bacteria associated with the highly virulent *B*. *xylophilus* AmA3 were *Stenotrophomonas* and Rhizobiaceae_Unclassified. The predominant bacteria associated with moderately virulent *B*. *xylophilus* AA3 were *Stenotrophomonas* and *Achromobacter*. The predominant bacteria associated with moderately virulent *B*. *xylophilus* strain HE2 were Rhizobiaceae_Unclassified, *Achromobacter* and *Luteibacter*.

**Table 4:** The sorting of PWN-associated bacteria according to the relative abundance.

	The abundance of nematode associated bacteria				
Nematode strains	1	2	3	4	5
ZL1 (HV)	Pseudomonadaceae_Unclassified (35.9%)	*Pseudomonas* (19.7%)	*Stenotrophomonas* (12.4%)	*Achromobacter* (6.6%)	Rhizobiaceae_Unclassifie (6.1%)
AmA3 (HV)	*Stenotrophomonas* (35.9%)	Rhizobiaceae_Unclassifie (24.4%)	*Chitinophaga* (8.2%)	Oxalobacteraceae_Unclassified (4.5%)	*Ochrobactrum* (2.5%)
AA3 (MV)	*Stenotrophomonas* (48.7%)	*Achromobacter* (9.2%)	Enterobacteriaceae_Unclassified (4.2%)	Oxalobacteraceae_Unclassified (2.9%)	*Escherichia* (2.9%)
HE2 (MV)	Rhizobiaceae_Unclassified (40.2%)	*Achromobacter* (16.6%)	*Luteibacter* (16.5%)	*Stenotrophomonas* (3.7%)	Pseudomonadaceae_Unclassified (2.7%)
CFS1 (MV)	Pseudomonadaceae_Unclassified (31.2%)	Rhizobiaceae_Unclassified(10.1%)	*Stenotrophomonas* (9.5%)	Oxalobacteraceae_Unclassified (7.4%)	Sphingobacteriaceae_Unclassified (6.3%)
SD1 (MV)	*Stenotrophomonas* (27.7%)	Pseudomonadaceae_Unclassified (24.2%)	Oxalobacteraceae_Unclassified (14.7%)	Sphingobacteriaceae_Unclassified (5.1%)	Enterobacteriaceae_Unclassified (4.5%)
JNL10 (LV)	Oxalobacteraceae_Unclassified (23.3%)	*Achromobacter* (19.5%)	Pseudomonadaceae_Unclassified (10.9%)	Rhizobiaceae_Unclassifie (9.7%)	*Pseudomonas* (6.2%)
GHB3 (NV)	*Stenotrophomonas* (21.5%)	Oxalobacteraceae_Unclassified (12.1%)	Rhizobiaceae_Unclassified(10.1%)	Sphingobacteriaceae_Unclassified (9.4%)	Enterobacteriaceae_Unclassified (8.2%)

#### Predominant bacteria associated with *B*. *mucronatus*

As shown in Table 4, the predominant bacteria associated with moderately virulent *B*. *mucronatus* CFS1 were Pseudomonadaceae_Unclassified and Rhizobiaceae_Unclassified. The predominant bacteria associated with moderately virulent *B*. *mucronatus* SD1 were *Stenotrophomonas* and Pseudomonadaceae_Unclassified. The predominant bacteria associated with the lowly virulent *B*. *mucronatus* JNL10 were Oxalobacteraceae_Unclassified and *Achromobacter*. Meanwhile, the predominant bacteria associated with the non-virulent *B*. *mucronatus* GHB3 were *Stenotrophomonas* and Oxalobacteraceae_Unclassified.

#### Community structures of bacterial associated with *B*. *xylophilus* and *B*. *mucronatus* with different virulence

**Fig 4:**
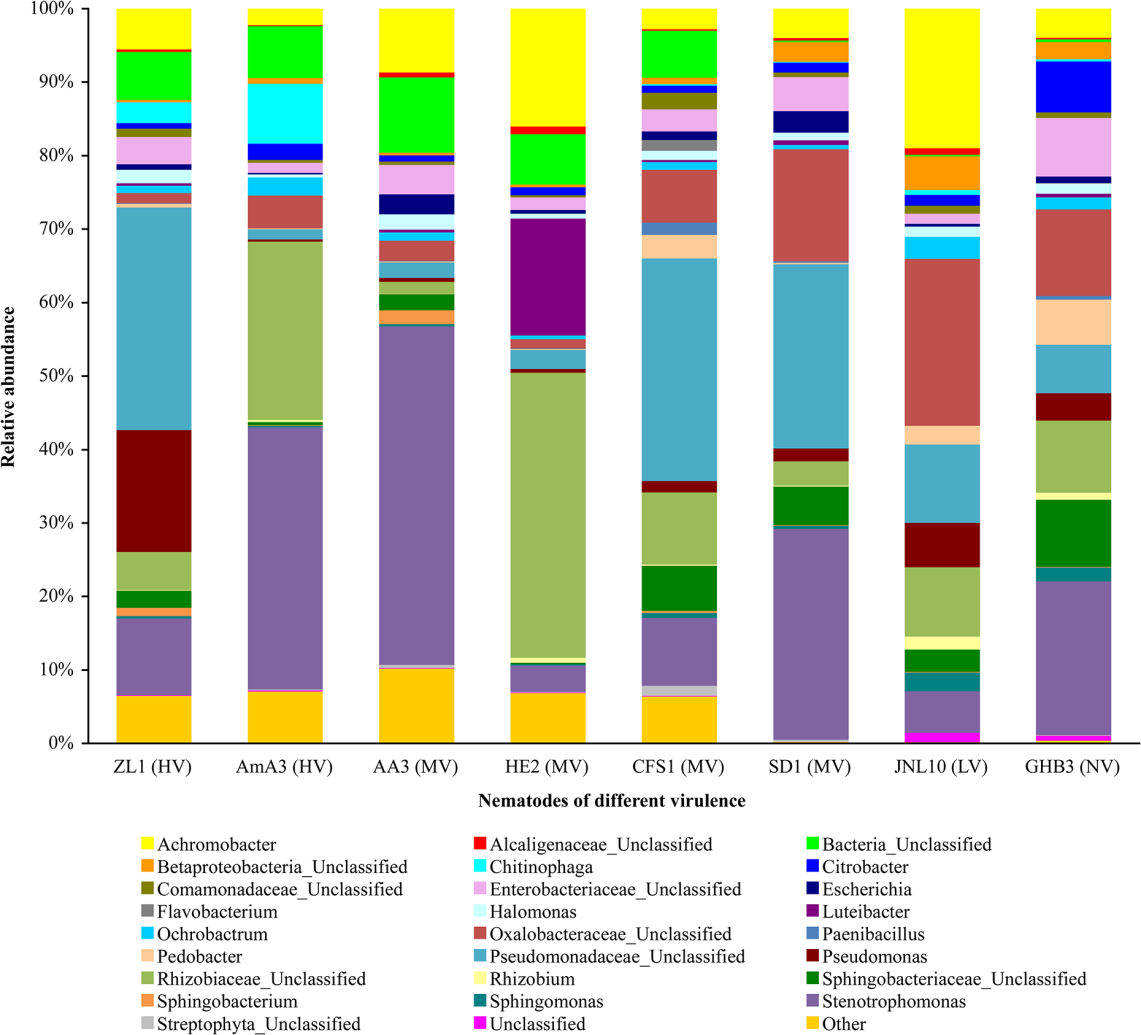
The bacterial composition of *B*. *xylophilus* and *B*. *mucronatus* at genus level.

As well as the species and abundance of predominant bacteria associated with the nematodes which had different virulence were different, and the bacteria associated with each nematode were different. *Stenotrophomonas*, Rhizobiaceae_Unclassified, *Achromobacter*, Enterobacteriaceae_Unclassified and Pseudomonadaceae_Unclassified occupied large proportions of the bacteria associated with the nematodes (Fig 4). However, the proportions were different. The abundance of other associated bacteria, for example, Streptophyta_Unclassified, *Sphingomonas*, *Sphingobacterium*, *Rhizobium*, *Paenibacillus*, *Flavobacterium*, *Escherichia* and Comamonadaceae_Unclassified, were similar in *B*. *xylophilus* and *B*. *mucronatus* with different virulence.

The abundance of Betaproteobacteria_Unclassified, Sphingobacteriaceae_Unclassified, Oxalobacteraceae_Unclassified and *Pedobacter* in *B*. *mucronatus* was significantly higher than those in *B*. *xylophilus*. The abundance of Bacteria_Unclassified associated with *B*. *xylophilus* and virulent *B*. *mucronatus* strain CFS1 were significantly higher than those in *B*. *mucronatus* strain SD1, JNL10 and GHB3. The proportions of Pseudomonadaceae_Unclassified in highly virulent *B*. *xylophilus* strain ZL1 and moderately virulent *B*. *mucronatus* strain CFS1 and SD1 were significantly higher than those in other nematodes. The proportions of *Chitinophaga* in highly virulent *B*. *xylophilus* strain ZL1 and AmA3 were 3.4% and 8.2%, respectively. However, there were no *Chitinophaga* in moderately virulent *B*. *xylophilus* strain AA3 and strain HE2. Only a few *Chitinophaga* existed in the bacteria associated with *B*. *mucronatus*, and the proportions were less than 0.7%. The abundance of *Sphingomonas* in lowly virulent *B*. *mucronatus* strain JNL10 and non-virulent *B*. *mucronatus* strain GHB3 were 2.6% and 1.9%, respectively, and they were significantly higher than those in other nematodes. *Achromobacter* occupied a certain proportion in all of the nematode-associated bacteria, and the proportions were relatively high in moderately virulent *B*. *xylophilus* strain AA3 and non-virulent *B*. *mucronatus* strains JNL10 and GHB3 (Fig 4).

## Discussion

### Advantages of 16S rDNA high-throughput sequencing technology

In this study, the diversities of bacteria associated with *B*. *xylophilus* and *B*. *mucronatus* with different virulence were analyzed by 16S rDNA high-throughput sequencing. Comparing to traditional culture method, it enabled us to analyze unculturable bacteria. Twenty four groups of nematode-associated bacteria with different virulence, such as *Stenotrophomonas*, Pseudomonadaceae_Unclassified and Rhizobiaceae_Unclassified, had relatively high abundance. This indicated that the species of bacteria associated with *B*. *xylophilus* and *B*. *mucronatus* were very abundant. Wu et al. isolated 15 endo-bacteria from *B*. *xylophilus* including *Stenotrophomonas* and *Achromobacter* [26]. *Stenotrophomonas maltophilia* and *Myroides* were isolated from *B*. *mucronatus* [44]. Through the construction of clone libraries and 454 sequencing, 21 genera of bacteria were associated with *B*. *xylophilus* were found [44]. Bacterial strains were grouped into 38 RAPD-types on basis of visual similarities, these bacterial strains belonged to 16 genera [39]. While only a small number of culturable bacteria could be obtained from the culture-depended methods [26, 34]. Therefore, compared to these techniques, the bacteria associated with *B*. *xylophilus* and *B*. *mucronatus* obtained by 16S rDNA high-throughput sequencing were more abundant. Bacteria communities isolated and identified by different methodologies from the nematodes occurring in USA, Japan, China, Korea and Portugal were considerably diverse and ubiquitous [28]. In comparison with the afore-mentioned the same predominant species in *B*. *xylophilus* and *B*. *mucronatus*, the new phyla found was Chloroflexi, Cyanobacteria, Gemmatimonadetes, Planctomycetes/Verrucomicrobia. Although we could not exclude the possibility that a trace number of ecto-bacteria would remain in the sample of bacterial community for high throughput sequencing, we deduce that the major component analyzed should be endo-bacteria, because most of ecto-bacteria would be removed in the course of multiple rinsing.

### The virulence of *B*. *xylophilus* and *B*. *mucronatus* remained stable in the long-term subculture

Large differences of virulence were found between *B*. *xylophilus* and *B*. *mucronatus* strains [16–18]. The pathogenicity of nematodes in this study showed that all *P*. *thunbergii* seedlings inoculated with the four *B*. *xylophilus* strains were dead. The disease development and dying process of *B*. *xylophilus* strains ZL1 and AmA3 took shorter time than the strains AA3 and HE2. The result was consistent with that by Liu (2007), who determined that the *B*. *xylophilus* ZL1 and AmA3 were high virulent strains, and AA3 and HE2 were moderately virulent strains [45]. Measurement of the pathogenicity of *B*. *mucronatus* showed that the strains CFS1 and SD1 were pathogenic on *P*. *thunbergii* seedlings, and all of the seedlings were infected after inoculation by the two strains. However, the *B*. *mucronatus*’ infection of *P*. *thunbergii* seedlings was lower than those inoculated with *B*. *xylophilus*, and the wilt symptoms took longer time to develop. Thirty one days after *P*. *thunbergii* seedlings inoculated with *B*. *mucronatus* JNL10, the wilt symptoms were observed for the first time. The disease severity index was very low. These results indicated that the pathogenicity of *B*. *mucronatus* JNL10 was weak, and the GHB3 strain was not pathogenic, which were consistent with previous reports [26]. Our findings indicated that the virulence of *B*. *xylophilus* and *B*. *mucronatus* remained stable in the long-term subculture.

### The diversity of bacteria from *B*. *xylophilus* and *B*. *mucronatus* with different virulence

Many studies suggested that the pathogenicity of *B*. *xylophilus* was related with the associated bacteria [27–28, 31]. In our study, some OTUs were annotated as Bacteria_Unclassified in the sequencing results, indicating that some species of bacteria associated with *B*. *xylophilus* and *B*. *mucronatus* could not be identified. The abundances of Bacteria_Unclassified in the four *B*. *xylophilus* strains and virulent *B*. *mucronatus* strain CFS1 were significantly higher than those in *B*. *mucronatus* strains SD1, JNL10 and GHB3. The richness indexes of highly virulent *B*. *xylophilus* strains ZL1 and AmA3 were significantly higher than those of other nematode strains. This indicated that the higher virulence a nematode had, the more richness. It suggested that the adaptability of high virulence *B*. *xylophilus* to ecological environment may be better. The study also found high virulence *B*. *xylophilus* ZL1 and AA3 had different associated-bacterial compositions. Although the two strains were belonged to high virulent nematode and isolated from the same pine species. However, the sampling places were different, one from Zhejiang and another from Anhui. It suggested that the bacteria were associated with the sampling geographic difference, soils, pine species and vectors. Thus, the differences found in these two high virulent strains would be reasonable.

Tian [44] made a comparison of community differences of the associated bacteria between M- and R-type *B*. *xylophilus*. Certain bacteria, such as *Stenotrophomonas* and *Pseudomonas* existed among the bacteria associated with R-type *B*. *xylophilus* and were absent among the ones with the M-type. In our study *Stenotrophomonas* or Pseudomonadaceae_Unclassified or Rhizobiaceae_Unclassified were found in the bacteria associated with virulent *B*. *xylophilus* and *B*. *mucronatus*. The afore-mentioned bacteria generally exist in *B*. *xylophilus* and *B*. *mucronatus* with high levels of relative abundances. The results indicated that the three kinds of bacteria mentioned above were predominant in the nematode-associated bacteria. Meanwhile, the proportions of *Chitinophaga* in highly virulent *B*. *xylophilus* strain ZL1 and AmA3 were significantly higher than other strains. Oxalobacteraceae was more abundant in the bacterial communities associated with lowly virulent *B*. *mucronatus* strain JNL10 and non-virulent *B*. *mucronatus* strain GHB3. The amount of *Achromobacter* was relatively high in moderately virulent *B*. *xylophilus* strain AA3, HE2 and non-virulent *B*. *mucronatus* strains JNL10 and GHB3. This suggested that the above PWN-associated bacteria may be related to the virulence of the nematodes. Our study also found the differences of abundance of other eight genera of bacteria associated with *B*. *xylophilus* and *B*. *mucronatus* with different virulence, such as *Sphingomonas*, were not significant. This suggests that these bacteria existed generally in the nematode strains and may have no direct relationship with the virulence of the nematodes. The evidence of these bacteria involved in the pathogenic processes of *B*. *xylophilus* need to be further studied.
